# Appropriateness of American Society for Gastrointestinal Endoscopy Guidelines for Upper Gastrointestinal Endoscopy: A Prospective Analytical Study

**DOI:** 10.7759/cureus.4062

**Published:** 2019-02-13

**Authors:** Susan Rajan, Anandhi Amaranathan, Subitha Lakshminarayanan, Sathasivam Sureshkumar, Manoj Joseph, Vishnu Prasad Nelamangala Ramakrishnaiah

**Affiliations:** 1 Surgery, Jawaharlal Institute of Postgraduate Medical Education and Research (JIPMER), Puducherry, IND; 2 Preventive Medicine, Jawaharlal Institute of Postgraduate Medical Education and Research (JIPMER), Puducherry, IND; 3 Biochemistry, Jawaharlal Institute of Postgraduate Medical Education and Research (JIPMER), Puducherry, IND

**Keywords:** upper gastrointestinal endoscopy, asge guidelines, appropriateness of endoscopies, indications for endoscopies

## Abstract

Introduction

Extensive use of upper gastrointestinal endoscopy (UGE) with the advent of open access centers has resulted in inappropriate endoscopies. Our study aimed to evaluate the appropriateness of American Society for Gastrointestinal Endoscopy (ASGE) guidelines for UGE and to assess the diagnostic yield of endoscopy in a tertiary care center in South India.

Methods

The study was conducted as a prospective analytical study. Indications for endoscopy were classified as “ASGE appropriate” and “ASGE inappropriate”. The significance of association of ASGE guidelines and other categorical variables with endoscopic findings were assessed.

Results

ASGE appropriate indications and inappropriate indications accounted for 85.9% and 14.1% of endoscopies, respectively. The most common appropriate indication was persistent dyspepsia despite adequate proton-pump inhibitor (PPI) therapy (28.1%) and the only inappropriate indication for endoscopy was isolated dyspepsia without adequate PPI therapy (14.1%). The diagnostic yield of endoscopy for appropriate indications was 69.5% and for inappropriate indications was 55.1%, the difference was statistically significant (P= 0.003; OR-1.857). The sensitivity and specificity of ASGE guidelines was 88.5% and 19.5%, respectively.

Conclusion

According to our study, ASGE guidelines may be considered as appropriate guidelines for UGE in our population and these guidelines were followed 85.9% of the times in referring patients for the same. However, the high diagnostic yield even in inappropriate endoscopies indicates the necessity of further studies that might identify other relevant indications for endoscopy, thus avoiding misutilization of resources without missing out on relevant cases.

## Introduction

Use of upper gastrointestinal endoscopies (UGEs) for diagnosis, treatment, surveillance or exclusion of gastroduodenal diseases led to the advent of open access endoscopy where elective endoscopies are scheduled by general practitioners, without prior consultation with a specialist [1,2]. This has resulted in inappropriate endoscopies and overutilization of limited healthcare resources causing long waiting times for endoscopy at many health centers, resulting in delayed intervention in many cases with serious pathology [3,4].

In order to frame guidelines for the use of endoscopy, many professional bodies have conducted studies and evaluated the diagnostic yield of various gastrointestinal symptoms and signs [5-11]. However, the appropriateness of these guidelines has not been universally proven. Studies are still being conducted worldwide to assess the appropriateness of various guidelines and to produce a universally acceptable set of guidelines [12-17]. One of the more widely used guidelines is the American Society for Gastrointestinal Endoscopy (ASGE) guidelines used by many endoscopic centers in India, but they have been formulated on the basis of studies done in the Caucasian population and the appropriateness of these guidelines in the Indian population is yet to be evaluated.

Hence, we performed this study in order to evaluate the appropriateness of ASGE guidelines (published in 2012) [9] for upper gastrointestinal endoscopy in the Indian population in a tertiary hospital in South India. This study also assesses the diagnostic yield of endoscopy in both appropriate indications and inappropriate indications.

## Materials and methods

This study was conducted as a prospective analytical study in the endoscopy unit of the Department of Surgery from October 2015-April 2017. The study commenced after getting approval from the institute ethical committee. Subjects were enrolled based on inclusion and exclusion criteria after taking informed consent. The study population included all patients more than 18 years of age who were referred to the endoscopy unit of the Department of Surgery during the study period. Patients who had already undergone UGE in the past and had a definitive diagnosis, patients who had previous therapeutic UGE or surgical treatment for any upper gastrointestinal conditions, and UGE abandoned due to any reason (inadequate preparation, uncooperative patient) were excluded from the study. Subjects were recruited based on a systematic random sampling of endoscopy clinic days to avoid bias. Based on 47% prevalence of abnormal findings on UGE [18], with an absolute precision of 5%, power of 80%, and an alpha error of 5%, the sample size was calculated to be 661 (95% confidence interval). The study was registered with the clinical trial registry of India with a registration number of CTRI/2018/02/011903.

Data collected from the study participants were recorded in a predesigned data collection sheet. The variables collected include independent variables like name, age, gender, body mass index (BMI), indications for endoscopy and details of the patient’s symptoms including presence of alarm symptoms (age > 50 years with new onset symptoms or signs suggesting structural disease, family history of upper GI malignancy, gastrointestinal bleeding or anemia, progressive dysphagia or odynophagia, persistent vomiting, unintended weight loss), treatment with proton pump inhibitors (PPI), comorbidities, alcohol and tobacco use, and use of gastric irritant drugs like non-steroidal anti-inflammatory drugs (NSAIDs). The indications for endoscopy were then classified as “ASGE appropriate” and “ASGE inappropriate” based on the ASGE guidelines.

The outcome variable was positive endoscopic findings, which included erosive esophagitis, erosive gastritis, erosive duodenitis, esophageal varices, gastric varices, duodenal ulcer, gastric ulcer, esophageal tumor, gastric tumor, gastric polyp, strictures, hiatal hernia, Barrett’s esophagus, and other relevant endoscopic findings. Details of any procedures performed during UGE were also recorded. Urease test was done using the rapid urease test and the final reading of the test was done at 24 hours and recorded.

Statistical analysis

Statistical analysis was performed using the Statistical Package for the Social Sciences (SPSS) software (Version 19.0) (IBM Corp., Armonk, NY, USA). Categorical data such as age, gender, indications for doing an endoscopy, the frequency of each individual indication, and relevant endoscopic findings were expressed as proportions. Continuous variables like age and BMI were expressed as mean with standard deviation (SD).

Appropriateness of endoscopy was calculated based on the ASGE guidelines and was expressed in percentage. The diagnostic yield of endoscopy was calculated in terms of the frequency of positive endoscopic findings for ASGE guidelines. The significance of the association of ASGE guidelines, individual indications, and other categorical variables with endoscopic findings was assessed using the chi-square test and odds ratios (OR) with 95% confidence intervals (CI). The association of relevant endoscopic findings and appropriate and inappropriate indications was also expressed as the odds ratio with 95% confidence intervals. The sensitivity and specificity of the ASGE guidelines and each individual indicator of endoscopy were also calculated. All statistical analysis was carried out for two-tailed significance and a P value ≤ 0.05 was considered as statistically significant. Multivariate analysis was done using binary logistic regression with endoscopic indications and categorical variables as independent variables and endoscopic finding as the dependent variable, and adjusted odds ratios were calculated.

## Results

A total of 757 subjects were included in the study of whom 57.2% (n=433) were males and 42.8% (n=324) were females. The mean age of the study subjects was 47 years (± 14.64) with 43.3% (n=290) of the subjects having age more than 50 years. The baseline and clinical characteristics of the study population are listed in Table 1.

**Table 1:** Association of baseline and clinical characteristics of the study subjects with positive endoscopic findings *Chi-Square Test; 95% CI – 95% Confidence Interval; NSAID – Non-Steroidal Anti-Inflammatory Drugs; BMI – Body Mass Index

S. No.	Study Characteristics		Overall % (N=757)	Positive Endoscopic Finding % (N = 511)	Odds Ratio (95% CI)	P-value*
1.	Age	>50 years	43.3	71.0	1.332 (0.977-1.817)	0.070
		≤49 years	56.7	64.8	-	
2.	Gender	Male	57.2	72.5	1.701 (1.251-2.312)	0.001
		Female	42.8	60.8	-	
3.	Comorbidities	Present	18.8	64.1	0.808 (0.565-1.215)	0.335
		Absent	81.2	68.3	-	
4.	NSAID or another drug use	Present	4.5	58.8	0.675 (0.335-1.360)	0.269
		Absent	95.5	67.9	-	
5.	Tobacco use	Present	40.2	73.7	1.620 (1.177-2.228) -	0.003
		Absent	59.8	63.4		
6.	Alcohol use	Present	49.5	73.9	1.788 (1.313-2.435)	<0.001
		Absent	50.5	61.3	-	
7.	BMI	Underweight	7.5	78.9	1.740 (0.771-3.927)	0.183
		Overweight & obese	45.7	61.8	0.656 (0.420-1.027)	0.065
		Normal	44.9	71.5	-	-

The indication for endoscopy was ASGE appropriate in 85.9% (n=650) and ASGE inappropriate in 14.1% (n=107) of endoscopies (Table 2).

**Table 2:** Association of the ASGE guidelines with positive endoscopic findings (n = 757) *Chi-Square Test; 95% CI – 95% Confidence Interval; ASGE – American Society for Gastrointestinal Endoscopy

S. No.	ASGE Guidelines	Overall % (N=757)	Positive Endoscopic Finding % (N=511)	Odds Ratio (95% CI)	P-value*
1.	Appropriate	85.9	452 (69.5%)	1.857 (1.25-2.815)	0.003
2.	Inappropriate	14.1	59 (55.1%)	-	

The most common appropriate indication for UGE was persistent dyspepsia despite adequate PPI therapy, accounting for 28.1% (n=213) of all cases. Other common ASGE appropriate indications were gastrointestinal bleeding or anemia (18.1%) and patients with age more than 50 years with new onset symptoms or upper abdominal symptoms or signs suggesting structural disease (16.6%). The only indication for endoscopy in our study that was ASGE inappropriate was isolated dyspepsia without an adequate trial of PPI in patients under the age of 50 years, which accounted for all the ASGE inappropriate endoscopies (Table 3).

**Table 3:** Indications for endoscopy and its association with positive endoscopic finding (n=757) *Chi-Square Test; 95% CI – 95% Confidence Interval; ASGE – American Society for Gastrointestinal Endoscopy; PPI – Proton-Pump Inhibitor; GI – Gastrointestinal; 2.1% of ASGE appropriate indications were for other indications like poisoning, foreign body ingestion, etc.

S. No.	Indications for Endoscopy	Overall % (N=757)	Positive Endoscopic Finding% (N=511)	Odds Ratio (95% CI)	P-value*
I	ASGE appropriate indications				
1	Persistent dyspepsia despite adequate PPI therapy	28.1	62.9	0.751 (0.539-1.048)	0.091
2	Age >50 years with new onset upper abdominal symptoms and signs	16.6	56.3	0.560 (0.379-0.828)	0.003
3	GI bleeding or anemia	18.1	82.5	2.626 (1.641-4.202)	<0.001
4	Progressive dysphagia & odynophagia	12.5	81.1	2.247 (1.313-3.847)	0.003
5	Persistent vomiting	6.6	84.0	2.664 (1.231-5.765)	0.010
6	Unintended weight loss	1.6	83.3	2.435 (0.529-11.200)	0.238
7	Family history of upper GI malignancy	0.3	50.0	0.480 (0.030-7.712)	0.545
II	ASGE inappropriate indications				
1	Isolated dyspepsia without adequate PPI therapy	14.1	55.1	0.538 (0.355-0.816)	0.003

The diagnostic yield of UGE was measured as the frequency of positive endoscopic findings. Positive endoscopic findings were found in 67.5% (n=511) of the study population. The most common findings were erosions (37.4%), followed by mass (12%), ulcer (7.4%), and varices (4.6%). The diagnostic yield of endoscopy for appropriate indications was 69.5% (452/650) while the same was 55.1% (59/107) in inappropriate indications (Table 2). The most common finding in both groups were erosions accounting for 36.9% in ASGE appropriate indications and 40.2% in ASGE inappropriate indications.

A biopsy was taken for rapid urease test in subjects in whom H. pylori disease was suspected. The urease test was performed in only 177 of study subjects with a positive test reported in 28.3% (50) and treated with anti H-pylori regimen. A biopsy for histopathological examination was performed in subjects with newly detected mass lesions or suspicious ulcers. A biopsy was performed in 102 subjects in which 84 cases of malignancy were diagnosed. Of these 84 new malignancies, three cases were detected among the patients in whom endoscopy was not indicated as per ASGE guidelines.

Relevant endoscopic findings were found to have a significant positive association with male gender, tobacco use, and alcohol consumption. Relevant findings were seen in 72.5% of males compared to 60.8% of females with an OR of 1.701 (95% CI = 1.251–2.312). We found that 73.7% of tobacco users (N=304) had relevant endoscopic findings compared to 63.4% of patients not using tobacco (N=453) with an OR of 1.620 (95% CI 1.17–2.228). In patients with a history of alcohol usage (N=375) relevant findings were seen in 73.9% compared to 61.3% in non-alcoholics (N=382) with OR of 1.788 (95% CI 1.313–2.435).

The difference in relevant endoscopic findings for appropriate and inappropriate indications (69.5% vs. 55.1%) was found to be statistically significant (p = 0.003; chi-square 8.68) with an odds ratio of 1.857 (95% CI = 1.225–2.815) (Table 2). Thus, in this study, it was seen that ASGE guidelines are appropriate guidelines for our study population in deciding the indications for endoscopy.

Of the appropriate indications evaluated, positive endoscopic findings were found to be significantly associated with GI bleeding (p<0.001), dysphagia (p=0.003), and persistent vomiting (p=0.010). The diagnostic yield of endoscopy in patients with GI bleeding, dysphagia, persistent vomiting, and unintended weight loss was >80% (82.5%, 81.1%, 84.0%, 83.3%, respectively). Even though erosions, ulcers, varices, and mass lesions were more frequently detected in subjects for whom endoscopy was ASGE indicated, only mass lesions were found to be statistically significant with an OR of 5.428 (95% CI of 1.685–17.482) (Table 4).

**Table 4:** Endoscopic findings according to the appropriateness of the indication (n=757) *Chi-Square Test; 95% CI – 95% Confidence Interval

S. No.	Endoscopic Finding	Appropriate Indication N (%)	Inappropriate Indication N (%)	Odds Ratio (95% CI)	P-value*
1	Erosions	240 (84.8%)	43 (15.2%)	0.871 (0.574-1.323)	0.520
2	Ulcer	51 (91.1%)	5 (5%)	1.737 (0.677-4.456)	0.245
3	Varices	34 (97.1%)	1 (2.9%)	5.851 (0.792-43.197)	0.050
4	Mass lesions	88 (96.7%)	3 (3.3%)	5.428 (1.685-17.482)	0.002
5	Stricture	7 (77.8%)	2 (22.2%)	0.572 (0.117-2.789)	0.371

With endoscopic examination as the gold standard, the sensitivity and specificity of ASGE guidelines were calculated. The sensitivity of ASGE guidelines was 88.5%, but the specificity was only 19.5%. Thus, ASGE guidelines are appropriate for the screening of upper GI conditions in our study population. The positive predictive value and negative predictive value of ASGE guidelines were 69.5 and 44.9, respectively, in this study. Among the individual ASGE appropriate indications, high specificity was seen in individual symptoms like unintended weight loss, GI bleeding, dysphagia, odynophagia and persistent vomiting, (99.2%, 90.2%, 92.7% and 96.7%, respectively), making these symptoms almost a mandatory indication for UGE. These symptoms also have a high positive predictive value of more than 80%, which correlates with the high diagnostic yield of these symptoms as mentioned earlier. However, low sensitivity (2%, 22.1%, 15.1%, 8.2%, respectively) of these individual symptoms makes them poor independent endoscopic indications (Table 5).

**Table 5:** Sensitivity, specificity, and predictive values of ASGE guidelines in the current study (n=757) *NPV – Negative Predictive Value; PPV – Positive Predictive Value; ASGE – American Society for Gastrointestinal Endoscopy; PPI – Proton-Pump Inhibitor; GI – Gastrointestinal

S. No.	Indications	Sensitivity	Specificity	PPV	NPV
1	ASGE guidelines	88.5%	19.5%	69.5%	44.9%
2	Persistent dyspepsia despite adequate PPI therapy	26.2%	67.9%	62.9%	30.7%
3	Age more than 50 years with new onset symptoms or upper abdominal symptoms or signs indicating structural disease	13.9%	77.6%	56.3%	30.3%
4	GI bleeding or anemia	22.1%	90.2%	82.5%	35.8%
5	Progressive dysphagia or odynophagia	15.1%	92.7%	81.1%	34.4%
6	Persistent vomiting	8.2%	96.7%	84.0%	33.7%
7	Unintended weight loss	2%	99.2%	83.3%	32.8%
8	Isolated dyspepsia without adequate PPI therapy	11.5%	80.5%	55.1%	30.5%

Multivariate logistic regression was done and ASGE indications were found to have independent significant association with endoscopic findings with an adjusted OR of 1.753 (1.128–2.725) and a p-value of 0.013 when adjusted to other factors like age more than 50 years, male gender, alcohol and tobacco use, all of which were not found to have an independent association with endoscopic findings (Table 6).

**Table 6:** Multivariate analysis of indications and clinical parameters with endoscopy (n=757) *OR – Odds Ratio; 95% CI – 95% Confidence Interval; ASGE – American Society for Gastrointestinal Endoscopy

S. No.	Clinical Parameters and Indications	Adjusted OR (95% CI)	P-value
1	Age> 50 years	1.059 (0.755-1.485)	0.740
2	Male gender	1.223 (0.756-1.978)	0.413
3	Alcohol use	1.364 (0.824-2.257)	0.227
4	Tobacco use	1.222 (0.839-1.781)	0.296
5	ASGE Indications	1.753 (1.128-2.725)	0.013

## Discussion

This study was performed to evaluate the appropriateness of ASGE guidelines as an indication for endoscopy in a sample of the Indian population in a tertiary hospital. Endoscopy was performed for ASGE inappropriate indications in 14.1% of the study subjects. Dyspepsia in patients with age less than 50 years who did not receive an empirical trial of proton pump inhibitors was the indication in all these subjects. Studies performed around the world have reported a rate of inappropriate endoscopies varying from 10% to 39% [4,12,14,15,19]. The wide variation in reported results may partly be attributed to the fact that various studies have selected different guidelines as the benchmark against which inappropriateness was assessed. The rate of appropriate endoscopies in our study is high, which may be explained by the fact that our hospital is a tertiary center and patients are referred to the Surgery Endoscopy Unit only by the consultants and residents of the Surgery Department. Patients referred for endoscopy from other departments and outside hospitals are initially screened by the surgical department before doing an endoscopy. Many studies have found that specialists make fewer inappropriate referrals when compared to primary care physicians [8,12-14]. The role played by the health care setup of the study area has been highlighted in some studies. Higher rates of the inappropriateness of endoscopy have been reported from areas where open access endoscopy is prevalent and low rates are seen in tertiary care centers [4,12].

The sensitivity and specificity of ASGE guidelines for relevant endoscopic findings in our study were 88.5% and 19.5%, respectively. This high sensitivity and low specificity of ASGE guidelines are similar to that seen in literature so far [16,17]. The low specificity for ASGE guidelines loses significance considering the high rates of positive findings in inappropriate indications. Relevant endoscopic findings were seen in 55.1% of inappropriate indications compared to 69.5% of appropriate indications. The most common endoscopic finding among the inappropriate indications was erosions, accounting for 40.2%, followed by ulcer (4.7%). Three cases of upper GI malignancy were identified by endoscopy among the subjects for whom endoscopy was deemed inappropriate as per ASGE guidelines.

High rates of positive endoscopic findings have been observed in previous studies also. Studies conducted in Saudi Arabia by Azzam et al. and Aljibreen et al. found high frequencies (46% and 66.1%, respectively) of positive endoscopic findings in the patients with inappropriate indications [8,13]. The range of frequency of relevant findings in inappropriate indications in studies conducted in Europe, Asia, and the USA lies between 25% and 40% [12,15,20,21]. High yield of endoscopy among appropriate and inappropriate indications in our study is probably because all endoscopic abnormalities including erosions, hiatal hernia, and esophagitis are considered relevant in our study whereas other studies have classified some endoscopic findings like hiatal hernia, nonerosive gastritis, and duodenitis as non-relevant findings [14,15]. Other factors which are not assessed in this study may also contribute to this high diagnostic yield in ASGE inappropriate indications. Even though significantly higher rates of positive endoscopic findings are seen with appropriate indications in comparison with inappropriate indications, the high frequency of positive findings in indications judged inappropriate makes it difficult for these indications to be ignored. Some studies have shown that strictly following the existing guidelines may result in the surgeon missing out on cases of upper GI malignancy and other upper GI disorders [15,16]. Therefore, guidelines for endoscopy need to be tailored according to the population and its characteristics such as the prevalence of upper GI malignancy. Such customized guidelines might enable the healthcare system to identify more pathologies in its earlier stage in patients not otherwise meeting strict criteria for endoscopy.

The most common appropriate indication for endoscopy in our study was persistent dyspepsia despite adequate PPI therapy (28.1%), which is similar to recent literature from Asia and Europe [12,14,15] followed by GI bleeding or anemia (18.1%). The rate of referral for dyspepsia is on the rise with the advent of open access endoscopy units and increased health-seeking behavior in the younger age groups. But not all cases of dyspepsia in the young require endoscopy as many of them are functional and are not associated with any organic abnormality. Hence, recent studies and guidelines advocate evaluation of isolated dyspepsia in young before primary endoscopy by ‘test and treat’ regimen [22] (testing for H.pylori by a non-invasive method and treat if positive), which is not followed in our institute. Rather, young patients with isolated dyspepsia are initially given empirical therapy with PPI and those who still have persistent symptoms undergo endoscopy. In our study, subjects suspected to have acid peptic disease due to H. pylori infection during endoscopy underwent the rapid urease test. This was done at the discretion of the surgeon performing the endoscopy. No fixed guidelines were followed. Those who tested positive were given treatment with the 'omeprazole 20 mg bd + clarithromycin 500 mg bd + amoxicillin 1 g bd' (OCA) regimen.

In our study, the only inappropriate indication was “isolated dyspepsia without adequate PPI therapy” (14.1%) similar to many other studies [5,12]. Even though dyspepsia in patients under 50 years who have not received adequate PPI trial is not indicated for endoscopy, many studies have shown that even negative endoscopy in these patients significantly reduces the number of consultations and improves the quality of life. Patient satisfaction after an endoscopy for dyspepsia is higher as the exclusion of a serious disease provides reassurance to the patients and aids in the long-term management of the patients [23-25]. In our study, we used a cutoff age of 50 years as per the recent ASGE guidelines published in 2012 [9]. In the majority of other studies, the cutoff age limit is 45 years as per the previous edition of ASGE guidelines [5,12,16]. Other common inappropriate indications in previous studies include anemia without evidence of GI bleeding, functional symptoms, and surveillance of gastritis and esophagitis [12,14]. In our study, all cases of GI bleeding and cases of anemia that were referred to our endoscopy unit were screened by the consulting surgeons or surgical residents and were considered appropriate. Cases of surveillance and follow-up were excluded from our study.

The most common relevant endoscopic findings in our study were erosions both in subjects with appropriate and inappropriate endoscopies. The most common endoscopic finding in subjects with GI bleeding and anemia was ulcers, whereas the most common endoscopic finding in subjects with dysphagia and weight loss was a malignancy. The most common endoscopic finding in many previous studies was mucosal erosions [14,15]. Adang et al. also examined alarm symptoms as an indication for UGE and found the most common endoscopic finding in patients with anorexia and weight loss to be gastric cancer and for those with dysphagia to be esophageal cancer [5].

Many studies have evaluated several independent factors as indications for endoscopy. Some have shown that using age and alarm symptoms as indications for endoscopy is better than using cumbersome ASGE guidelines [5,16] whereas others have concluded that age and alarm symptoms do not predict the endoscopic findings among patients with dyspepsia [7]. Previous studies have shown that older patients have a higher rate of alarm symptoms and are the main indication for endoscopy. However, it’s also noted that using alarm symptoms alone as an indicator of endoscopy leads to a late diagnosis of malignancies [6]. In our study, the sensitivity of individual indications for endoscopy was poor, but most of them like GI bleeding, persistent vomiting, dysphagia, and odynophagia had high specificity. A similar finding was reported in a meta-analysis of the appropriateness of the indications for UGE [17]. Due to the high specificity of these alarm symptoms, they proposed the use of alarm symptoms in prioritizing the endoscopies in centers with a long waiting list to avoid delay in the diagnosis of malignancy.

The appropriateness of endoscopy in our study group was very high possibly because all cases that underwent endoscopy were initially screened by the consulting surgeons and surgical residents. Also, all endoscopic findings like erosions, esophagitis, non-erosive gastritis, etc., were included in the positive findings. Subjects with previous endoscopic diagnosis and follow-up endoscopies were not included in this study. Hence caution should be exercised while extrapolating these results to other centers.

## Conclusions

According to this study, ASGE guidelines may be considered appropriate for upper gastrointestinal endoscopy in the South Indian population. However, since the diagnostic yield is high in both appropriate and inappropriate indications, further studies are required to look for any other individual indication or parameter that will include the endoscopy positive cases in the inappropriate indication group. This will help in the proper utilization of resources without missing out on relevant cases like malignancies.

## References

[1] Keren D, Rainis T, Stermer E, Lavy A (2011). A nine-year audit of open-access upper gastrointestinal endoscopic procedures: results and experience of a single centre. Can J Gastroenterol.

[2] Mahajan RJ, Marshall JB (1997). Prevalence of open-access gastrointestinal endoscopy in the United States. Gastrointest Endosc.

[3] Leddin D, Armstrong D, Barkun AN (2008). Access to specialist gastroenterology care in Canada: comparison of wait times and consensus targets. Can J Gastroenterol.

[4] Froehlich F, Burnand B, Pache I (1997). Overuse of upper gastrointestinal endoscopy in a country with open-access endoscopy: a prospective study in primary care. Gastrointest Endosc.

[5] Adang RP, Vismans JF-JFE, Talmon JL, Hasman A, Ambergen AW, Stockbrügger RW (1995). Appropriateness of indications for diagnostic upper gastrointestinal endoscopy: association with relevant endoscopic disease. Gastrointest Endosc.

[6] Bowrey DJ, Griffin SM, Wayman J, Karat D, Hayes N, Raimes SA (2006). Use of alarm symptoms to select dyspeptics for endoscopy causes patients with curable esophagogastric cancer to be overlooked. Surg Endosc Interv Tech.

[7] Wallace M, Durkalski V, Vaughan J (2001). Age and alarm symptoms do not predict endoscopic findings among patients with dyspepsia: a multicentre database study. Gut.

[8] Azzam NA, Almadi MA, Alamar HH (2015). Performance of American Society for Gastrointestinal Endoscopy guidelines for dyspepsia in Saudi population: prospective observational study. World J Gastroenterol.

[9] Early DS, Ben-Menachem T, Decker GA (2012). Appropriate use of GI endoscopy. Gastrointest Endosc.

[10] Axon AT, Bell GD, Jones RH, Quine MA, McCloy RF (1995). Guidelines on appropriate indications for upper gastrointestinal endoscopy. Working Party of the Joint Committee of the Royal College of Physicians of London, Royal College of Surgeons of England, Royal College of Anaesthetists, Association of Surgeons, the British Society of Gastroenterology, and the Thoracic Society of Great Britain. BMJ.

[11] Vader JP, Froehlich F, Dubois RW (1999). The European Panel on the Appropriateness of Gastrointestinal Endoscopy (EPAGE): conclusion and WWW site. Endoscopy.

[12] Chan Y-M, Goh K-L (2004). Appropriateness and diagnostic yield of EGD: a prospective study in a large Asian hospital. Gastrointest Endosc.

[13] Aljebreen AM, Alswat K, Almadi MA (2013). Appropriateness and diagnostic yield of upper gastrointestinal endoscopy in an open-access endoscopy system. Saudi J Gastroenterol.

[14] Hassan C, Bersani G, Buri L (2007). Appropriateness of upper-GI endoscopy: an Italian survey on behalf of the Italian Society of Digestive Endoscopy. Gastrointest Endosc.

[15] Rossi A, Bersani G, Ricci G (2002). ASGE guidelines for the appropriate use of upper endoscopy: association with endoscopic findings. Gastrointest Endosc.

[16] Buri L, Hassan C, Bersani G (2010). Appropriateness guidelines and predictive rules to select patients for upper endoscopy: a nationwide multicenter study. Am J Gastroenterol.

[17] Di Giulio E, Hassan C, Marmo R, Zullo A, Annibale B (2010). Appropriateness of the indication for upper endoscopy: a meta-analysis. Dig Liver Dis.

[18] Trevisani L, Sartori S, Gilli G (2001). Appropriateness of upper gastrointestinal endoscopy: a hospital-based study. Dig Dis Sci.

[19] Bersani G, Rossi A, Suzzi A, Ricci G, De Fabritiis G, Alvisi V (2004). Comparison between the two systems to evaluate the appropriateness of endoscopy of the upper digestive tract. Am J Gastroenterol.

[20] Mahajan RJ, Barthel JS, Marshall JB (1996). Appropriateness of referrals for open-access endoscopy. How do physicians in different medical specialties do?. Arch Intern Med.

[21] Charles RJ, Chak A, Cooper GS, Wong RC, Sivak MV (1999). Use of open access in GI endoscopy at an academic medical center. Gastrointest Endosc.

[22] Shaukat A, Wang A, Acosta RD (2015). The role of endoscopy in dyspepsia. Gastrointest Endosc.

[23] Wiklund I, Glise H, Jerndal P, Carlsson J, Talley NJ (1998). Does endoscopy have a positive impact on quality of life in dyspepsia?. Gastrointest Endosc.

[24] Rabeneck L, Wristers K, Souchek J, Ambriz E (2003). Impact of upper endoscopy on satisfaction in patients with previously uninvestigated dyspepsia. Gastrointest Endosc.

[25] Lucock MP, Morley S, White C, Peake MD (1997). Responses of consecutive patients to reassurance after gastroscopy: results of self administered questionnaire survey. BMJ.

